# Pickled Eggs—And Preserving Jam

**Published:** 1915-07-24

**Authors:** 


Yours faithfully,

F. A. Hocking.

### Pickled Eggs—and Preserving Jam.

*To the Editor of* The Hospital.

Sir,—When I came to this institution there was a contract for eggs with the grocer at 1s. per dozen. I bought five dozen and thereby wasted 5s. At least two out of every dozen were impossible; the remainder left much to be desired. When I complained the reply was: "I am very sorry, but of course at that price I cannot guarantee them." I tried to get new-laid eggs from the farms, but they were very scarce and very dear, and so during that winter I had practically to cut out eggs from the dietary.

Since then each summer I have pickled large quantities. My method is exactly the same as that given by your correspondent in The Hospital of July 10. I have large earthenware vessels, each of which holds one gross of eggs. I get them from a farm daily warm out of the nest, and at once put them into the pickle. A label is attached to the vessel, with dates when the first and last eggs were put in, so as to ensure that the vessel first filled will be the first to be emptied.

I buy the eggs at one penny each, and while we are using them the price of fresh eggs in this district is twopence each. In this way I save many pounds to the institution, and have neither the heartbreak of rotten eggs nor the worry of securing fresh ones.

We have a garden wherein grow large quantities of gooseberries, raspberries, red and black currants, plums, damsons, and many other fruits. The institution is charged market price for all fruit and vegetables. Last year, on account of the rise in price of sugar, people considered it extravagant to make jam; and so in many districts fruit was left to rot. In this locality the price was very low, so instead of selling we preserved far more than the usual quantity, and the consequence is that, in spite of the high price which we paid for sugar, we are still using beautiful home-made jam at a lower price than the shop article can be procured at.—Yours faithfully,

July 15, 1915. Poor-Law Matron.

[These interesting experiences bear out the comment we appended to the letter from Mr. Alexander, the intensive egg-farmer, last week; that, namely, the desirability or otherwise of pickling eggs in summer depends first on the size of the institution (the largest not generally finding it worth while); and, secondly, on local conditions of the market. As to preserving jam, our correspondent's foresight would seem to have been well rewarded. Existing prices of fruit are still high, in spite of the recent rains, apparently because the local demands of camps throughout the country have disorganised the town and metropolitan markets. We would, however, commend to our correspondent the Note, published last week, on "A New Delicacy in Sick-Room Cookery."—Ed. The Hospital.]

